# The Bacteria's Guide to Survival

**DOI:** 10.1371/journal.pbio.0030140

**Published:** 2005-03-22

From *The Worst Case Scenario Survival Handbook*—with handy entries like “How to escape from killer bees” and “How to escape from quicksand”—to *The Zombie Survival Guide: Complete Protection from the Living Dead*, survival guides are one of the latest publishing fads.

If there was a market for it, a survival guide for bacteria might include topics like “How to use your pili to keep your host from going apoptotic.” A host's cells can respond to a bacterial infection with apoptosis, or programmed cell death. For bacteria that pass directly from host to host, this can pose a problem. If the bacteria are highly virulent and induce too much cell death, they could take down their host before they're able to jump ship, thus hurting the bacteria's chances of survival in the long run.

Earlier studies suggested that bacteria can use their pili, finger-like appendages that many bear on their surface, to pull on a host's cell membranes and thus influence the cell's behavior. But these studies, which looked at mutant bacteria that could not retract their pili, did not examine the matter of how the bacteria coax their hosts to stay alive.

Now, in *PLoS Biology*, a group of researchers present more direct evidence that bacteria can induce changes in hosts' gene expression—and possibly keep the host cells alive longer—through tiny tugs on cell membranes. The study, led by Magdalene So, examined gene activity in human epithelial cells infected with Neisseria gonorrhoeae, the bacteria responsible for the sexually transmitted disease gonorrhea.

By comparing cells infected with normal N. gonorrhoeae to those infected with a mutant strain with defective pili, the researchers found a subset of 52 host genes that had higher activity when the host was infected with the normal bacteria, suggesting that the pulls of the pili were responsible. They also ran a key control experiment with an artificial mechanical pull on the host cell membrane. By coating magnetic beads with a preparation of bacterial pili, the beads attached themselves to the cell membranes. Then, in the presence of a magnetic field, the beads tugged on the cell membrane, approximating the effects on gene expression during infection with normal bacteria.

Thus, the mechanical tugs seem responsible for triggering a signaling cascade in the host cells, which ultimately affects the host's gene expression. Many of the genes that increased in activity due to the tugs were already known to regulate apoptosis and cellular response to stress, including mechanical strain on the membrane. Also, a majority of these genes were known to be induced by a family of proteins called mitogen-activated protein kinases, or MAPKs. The researchers showed that blocking MAPKs reduced the activity of several of the genes that are usually enhanced by infection with the normal bacteria. Also, they found that cells infected with the bacteria tended to survive treatment with staurosporine, a chemical that normally induces apoptosis.

Overall, the group's findings support previous speculations that some bacteria influence gene expression and the fate of cells in their hosts by tugging on the host cells' membranes with their pili. For bacteria like N. gonorrhoeae that pass directly from host to host, the researchers argue, it would be in a bacterium's interest to help keep its host alive. And bacteria appear to do this with the help of their pili.

